# The presence of air sac nematodes in passerines and near-passerines in southern Germany

**DOI:** 10.1016/j.ijppaw.2023.05.004

**Published:** 2023-05-20

**Authors:** Salamatu Abdu, Melina Eisenring, Daniel Zúñiga, Gustavo Alarcón-Nieto, Heidi Schmid, Lucy M. Aplin, Hanja B. Brandl, Damien R. Farine

**Affiliations:** a Department of Biology, University of Konstanz, Universitätsstraße 10, 78464, Konstanz, Germany; b Department of Collective Behaviour, Max Planck Institute of Animal Behavior, Am Obstberg 1, 78315, Radolfzell, Germany; c Department of Evolutionary Biology and Environmental Studies, University of Zurich, Winterthurerstrasse 190, 8057, Zurich, Switzerland; d Universität Konstanz, Centre for the Advanced Study of Collective Behaviour, Universitätsstraße 10, 78464, Konstanz, Germany; e ETH Zurich, Department of Biology, Wolfgang-Pauli-Strasse 27, HIT F41, 8093, Zurich, Switzerland; f Department of Migration, Max Planck Institute of Animal Behavior, Am Obstberg 1, 78315, Radolfzell, Germany; g Cognitive and Cultural Ecology Research Group, Max Planck Institute of Animal Behavior, Am Obstberg 1, 78315, Radolfzell, Germany; h Division of Ecology and Evolution, Research School of Biology, Australian National University, 46 Sullivans Creek Road, Canberra, ACT, 2600, Australia; i Max Planck Institute of Animal Behavior, Am Obstberg 1, 78315, Radolfzell, Germany

**Keywords:** Air sac nematode, *Serratospiculoides amaculata*, Parasite, Host, Great tit, Infection

## Abstract

Major climatic changes in conjunction with animal movement may be associated with the spread of parasites and their vectors into new populations, with potentially important consequences for population persistence. Parasites can evolve to adapt to unsuitable ecological conditions and take up refuge within new host species, with consequences for the population growth of the new host species. One parasite species that has likely been increasing its geographic range, and potentially infecting new hosts, is the recently described air sac nematode *Serratospiculoides amaculata*, in great tits (*Parus major*) in Slovakia. In this study, we screened wild birds for potential air sac nematode infection in a woodland area of southern Germany. We identified four additional host species: Eurasian nuthatch, great spotted woodpecker, greenfinch and robin. As infection by this group of nematodes can be highly pathogenic, we recommend further investigation into its potential risk to these populations.

## Highlights

• Climatic changes and animal movement can aid the spread of parasites.
• These parasites can evolve to infect new host species.
• We report new cases of air sac nematode infection in avian species.

## Graphical abstract

**Figure:**
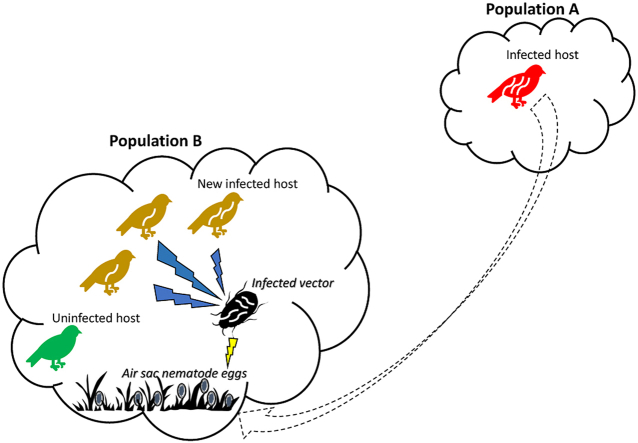

## 1 Introduction

Animal species are increasingly moving outside their ranges by shifting or expanding it in response to anthropogenic activities (Wilson et al., 2020) and climate change (Chen et al., 2011) with potential implications for host-parasite dynamics. Novel movement patterns and introduction of animals also provide opportunities for spread of other species, such as the parasites they harbor (Best et al., 2010; Kutz et al., 2005). For example, a malaria parasite was recently reported to have expanded its range to non-migratory birds in North America, likely due to spillover from introduced captive birds (Theodosopoulos et al., 2021). In another example associated with natural movements, the nomadic movement of cattle across regions in Asia and Africa has been linked with expanding distribution of ticks (Barré and Uilenberg, 2010). Climate warming may also enable parasites to become established in new geographical areas, where they can come into contact with new hosts (Kutz et al., 2004). New infections can then have consequences for the new host, such as negatively affecting clutch size, hatching success, and the number of young produced (Watson, 2013). Studies have suggested that parasites can have effects on populations similar to predation, with host-parasite interactions representing dominant links in interspecies interaction webs (Doi and Yurlova, 2011). In birds, nematodes have also been revealed to play a role in the death of adult and nestlings (Larrat et al., 2012; Wiese et al., 1977) Because parasites play a key role in the environment, it is paramount to monitor and track parasite population dynamics.

Birds are final hosts of filarid worms, also known as air sac nematodes. These air sac nematodes consist predominately of the genera *Diplotriaena*, *Serratospiculum* and *Serratospiculoides*, in the order Spirurida. As non-species-specific parasites, these nematodes are of biological importance because of their observable effects on the health of birds (Sonin, 1968). They cause air sacculitis, pneumonia and bronchopneumonia. Infections are associated with symptoms such as wheezing, coughing, dyspnea, loss of appetite, declining condition, lethargy and reduced flight performance (Sterner and Cole, 2009). Severe acute infection can lead to death, but also moderate disease can be accompanied by secondary infections such as *Salmonella* and avian influenza (Van Wettere et al., 2018). As symptoms are often not apparent, most of these infections are discovered incidentally in the wild when birds are examined for other purposes (Michalski et al., 2021), and systematic surveying of wild bird populations for new infections is rarely conducted.

To become established within an avian host, air sac nematodes must migrate successfully through the hepatic portal system. The eggs of the air sac nematodes must be consumed by a suitable intermediate host, such as an invertebrate—insects and other arthropods— to become infective and within this host, the eggs develop into a larval form. The intermediate host carrying the infective larvae then gets eaten by a bird, and after it is digested, the larvae emerge and penetrate the walls of the intestine into the blood stream where it develops further and migrates to the lungs to become a subadult (Cawthorn and Anderson, 1980; Samour and Naldo, 2001). Once in the lungs, it finally migrates to the air sac where it resides and eventually morphs into an adult worm. Adult male and female worms mate and reproduce sexually within the air sac, a gravid female then releases eggs intermittently, which are taken up into the trachea. From the trachea, embryonated eggs are coughed up and swallowed back into the gut where they are eventually excreted with faeces (Abdu et al., 2022; Samour and Naldo, 2001; Sterner and Cole, 2009).

Among the different species of air sac nematodes, *Diplotriaena* (subfamily Diplotriaeninae) are known to infect a wide range of birds, particularly passerines. However, *Serratospiculum* and *Serratospiculoides* (subfamily Dicheilonematinae) have—to date—been considered to be more specialized, primarily infecting falcons such as the prairie falcon *Falco mexicanus* and peregrine falcon *F. peregrinus* (Sonin, 1968). To our knowledge, these genera differ from each other mainly in their morphology, but little is known on how differently they affect the health of their hosts (Sonin, 1968). Over the years, new cases from these genera (and redocumentations) have been reported in corvids (Cawthorn and Anderson, 1980), starlings (Fasina et al., 2007), honeyeaters (Gosbell and Luk, 2019), kingfishers (Bronson et al., 2014), barbets (Webster and Speckmann, 1976), passerines (Sonin, 1974), and additional species of falcons (Santoro et al., 2016); spanning North America, Europe, Asia, Australia, and Africa. More recently, there have been efforts to create a database for the molecular sequences of these genera. For example, to corroborate the morphological characteristics of these species (Michalski et al., 2021; Stanicka et al., 2021), *Diplotriaena obtusa* has been sequenced in the blackcap *S. atricapilla* (in Europe), barn swallow *Hirundo rustica*, and cliff swallow *Petrochelidon pyrrhonota* (in the U.S.A.). However, these efforts remain relatively minor relative to the potential scale of movements of parasites across landscapes.

*Serratospiculoides amaculata* was first reported and sequenced in a parid host, the great tit *Parus major*, in Slovakia (Dolinská et al., 2018; Königová et al., 2013). While previously only known to infect falcons (Bain and Vassiliades, 1969; Schader and Baron, 2021; Van Wettere et al., 2018; Veiga et al., 2017), *S. amaculata* now appears to be expanding its hosts to passerines, and its geographic range, having recently been detected in great tits in southern Germany (Abdu et al., 2022). Common amongst some passerines is the near identical genus *Diplotriaena* (Seibert, 1944; Sonin, 1974), which has been discovered to infect blue tits *Cyanistes caeruleus* following a major mortality event in southern Germany (Rentería-Solís et al., 2021). However, *S. amaculata* infections may be present among other bird species but remain undetected. For instance, birds that were part of approved experiments and were euthanized due to health-related issues and subsequently dissected have been detected with parasites that were otherwise missed during faecal screening (S. Abdu, *pers. obs*.). Therefore, in this study we set out to scan for avian hosts that may have previously been missed, or were newly colonized, by screening faecal samples from wild bird species in southern Germany (n = 20 species, Table 1). Here, we describe a potential range/host expansion of the air sac nematode *S. amaculata* into four new bird species. We also highlight the importance of monitoring wild birds for the potential continual spread of parasites of biological importance—geographically and into new species. This will assist in the preparation and management of major disease outbreaks that may emerge owing to major global climatic changes.

**Table 1:** Total number of birds sampled per species and the number of samples tested positive for air sac nematode (*Serratospiculoides* spp.) eggs.

Species	No. Sampled	No. positive for air sac nematode
**Blackbird**	7	0
**Blue tit**	44	2
**Brambling**	1	0
**Bullfinch**	3	0
**Chaffinch**	7	0
**Coal tit**	1	0
**Crested tit**	1	0
**Dunnock**	1	0
**Eurasian nuthatch**	5	4
**Firecrest**	2	0
**Great tit**	46	9
**Great spotted woodpecker**	5	1
**Greenfinch**	11	1
**Goldcrest**	5	0
**Long-tailed tit**	4	0
**Marsh tit**	8	0
**Robin**	18	1
**Short-toed treecreeper**	1	0
**Song thrush**	1	0
**Wren**	1	0

## 2 Materials and methods

### 2.1 Study site

The study was carried out in the area surrounding the Max Planck Institute of Animal Behaviour (MPIAB) in Radolfzell (47.7452° N, 8.9669° E), Germany, between October 2020 and February 2022. Many passerines are found in this region, which is characterized by open fields and farmlands, forests and deciduous trees. Birds were captured in mist nets by HS (license DER-0702) as part of the long-term bird ringing program carried out by the Centre for Animal Marking of the MPIAB, or by GAN (license DER-1918) as part of projects under the licenses 55.8841.03 and 8853.17 issued by Regierungspräsidium Karlsruhe. Birds were trapped at a mixture of sites, and both with and without feeders to attract birds.

### 2.2 Sample collection

After having been extracted from the mist nets, birds were immediately transferred into clean, individual cloth bags, per regular field protocol. These bags were lined with sheets of paper to collect faecal samples. Most of the birds released faeces in the bags before they were further processed (fitting metal rings and recording biometrics). After birds were removed from the cloth bag for processing, any faeces on the paper lining was transferred immediately into a vial and stored in a cool place. Upon returning from the field, samples were stored in the refrigerator at 5 °C. If samples were not likely to be processed within a week, they were stored in 5% formaldehyde and kept at room temperature to preserve parasite eggs.

### 2.3 Faecal sample processing

Samples retrieved from birds were analyzed using the modified McMaster technique or direct smear – for samples weighing <0.1g (Greiner, 1989; Roepstorff and Nansen, 1998). Previous work comparing methods found that the McMaster technique provides the optimal balance in terms of processing time and performance at detecting the presence of air sac nematode eggs (Abdu et al., 2022). Parasites were identified to genus level with the aid of a binocular microscope (Leica DM500, Heerbrugg, Switzerland) at x100 and x400 magnification and a guide (Sonin, 1968). To determine the species of air sac nematode within the population, frozen faecal samples from an infected Eurasian nuthatch were sequenced after DNA extraction with the stool protocol from the NucleoSpin Tissue kit (Macherey-Nagel) and PCR amplification with Nematode 1/Nematode 2–28S rDNA pair primers and COlintF/ColintR mitochondrial cytochrome *c* oxidase subunit according to Binkienė et al. (2021). Although unknown nematodes were detected in these samples, DNA amplification failed, possibly due to inhibitors.

## 3 Results

**Fig. 1:**
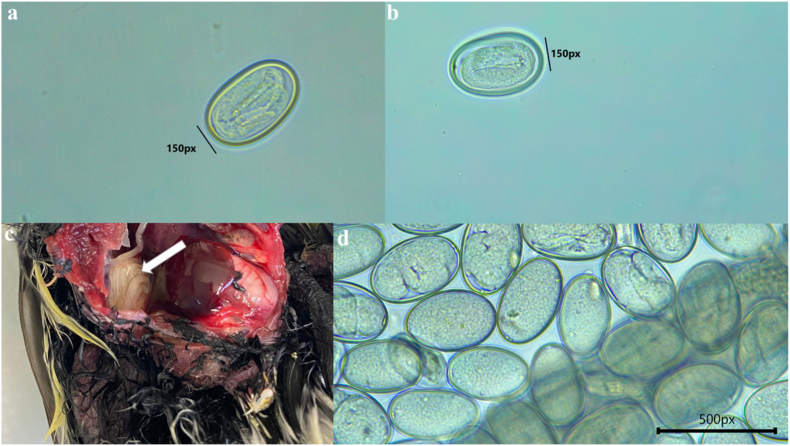
Embryonated air sac nematode eggs collected from a (**a**) Eurasian nuthatch and a (**b**) great tit faecal sample. A dead great tit with (**c**) adult worms within its air sac – white arrow – and (**d**) embryonated eggs from a female worm retrieved from the great tit in (c). Measurements are in pixels (px) and photos taken at X40.

We sampled a total of 172 birds, belonging to 20 species, for internal parasites. Of these, a total of 18 individuals across 6 species were positive for air sac nematode infection, representing 10% of the total number of birds sampled (Fig. 1; Table 1). Of these species, the Eurasian nuthatch, great spotted woodpecker, greenfinch and robin are avian hosts for *S. amaculata* that had not been previously reported. We found that 80% of the Eurasian nuthatches were positive for air sac nematode infection, with a range of 25–5375 eggs pers gram(epg). This was followed by 20% of the great tits (25–700 epg) and 5% of blue tits, with the remaining three other species having a single detection (Table 1). Dissection (performed by DZ) of a great tit that died during an experiment taking place at the same time was found to have adult air sac worms (Fig. 1c), some of which contained millions of eggs, and with the postmortem concluding that the air sac nematode was the likely cause of death. The air sac nematode eggs from all bird species were identical in morphology under the microscope—all the eggs found were oval in shape and embryonated (see Fig. 1). Other internal parasites identified within the samples included coccidia (such as *Isospora* sp.), tapeworms and other nematodes (such as *Capillaria* sp., *Syngamus* sp. and strongyles). The species infected with coccidia include blue tit, great tit, chaffinch, greenfinch and marsh tit. We also detected tapeworms in blackbird, blue tit, great tit, great-spotted woodpecker and robin samples, while, other nematodes species were found in blue tit, great tit, dunnock, long-tailed tit, robin and song thrush samples. Only six individuals across three species (robin, great-spotted woodpecker and great tit) had a coinfection.

## 4 Discussion

Our study extends the known prevalence of air sac nematodes in European passerines and near-passerines. Specifically, we report four previously undocumented avian host species infected with unknown air sac nematodes. About a third of all the species of birds sampled had at least one individual infected with an air sac nematode egg. This is an important finding as it may indicate either that the air sac nematodes are expanding their geographic range and might be colonizing a number of new avian host species, or that these parasites have remained undetected in these populations.

While it is difficult to distinguish between truly novel and undetected infections, we have good reasons to believe that our results point to the former. The unmistakable symptoms in the deceased great tit, together with extensive work done on great tits in the years leading up to its death, make it unlikely that the presence of the air sac nematode the population would have previously gone undetected. Further, in the following spring, several breeding birds were observed in nest-boxes seemingly very fatigued and wheezing (D. Farine, *pers. obs.*), something that had never been observed previously.

Our finding, that at least one third of species can host air sac nematode infections and a previous captive study on great tits recording a 37% prevalence of air sac nematodes with a maximum egg count of 10,800 per g (Abdu et al., 2022), is potentially significant in the context of recently described declines in passerines in Europe (Burns et al., 2021; Kamp et al., 2021; Rentería-Solís et al., 2021). While often attributed to changes in land use and habitat degradation, novel parasite infections may represent a hidden source of declines. Given the potentially high infection load we identified in the Eurasian nuthatch from this study and the great tit in Abdu et al. (2022), parasites are likely to be having major impacts. As all the birds in the present study were sampled in the field, and previous work has suggested that this could result in an underestimation of the true infection state because faeces collected at a single time-point can provide unreliable estimates of parasite abundance (Abdu et al., 2022), our results are likely to represent an low estimate of the true infection rates.

Unusual irruptive movements of woodland birds may play a role in cross-species host sharing; for example, a blue tit ringed in our population was recaptured 200 km SW in Switzerland, another was recaptured 600 km NE in Poland. In addition, a new migratory route adopted by black caps in recent decades may have assisted range expansion for potentially acquiring air sac nematode infection (Newton, 2007; Okulewicz and Sitko, 2012). Thus, air sac nematode species infecting specific avian families and orders may no longer be as restricted as previously thought. Some of the air sac nematodes may also be evolving to infect a variety of new intermediate and final hosts within native and new geographic ranges in addition to the known intermediate hosts likely to be present in new regions.

Based on previous studies (Rentería-Solís et al., 2021), it is likely that birds in this area are also infected with *D*. *obtusa.* We recommend that future studies look further into the molecular characterization of the air sac nematodes to species level for the avian hosts identified here. Morphological characterization of eggs can be difficult when utilizing non-invasive techniques such as the screening of faeces. However, faecal samples can be utilized as a method for regular monitoring during the banding of birds, allowing for potentially large-scale data collection. Finally, keeping up with the parasites present and their potential spread over time within the population will aid in understanding host-parasite dynamics, and help counter the potential risks of such highly pathogenic parasites to host populations. Such discoveries will also help to better understand the mechanisms surrounding the ecology and evolution of host-parasite systems.

## Declaration of competing interest

The authors whose names are listed immediately below certify that they have NO affiliations with or involvement in any organization or entity with any financial interest or non-financial interest in the subject matter or materials discussed in this manuscript.
